# Dr. Reed Brockway Bontecou

**Published:** 1907-05

**Authors:** 


Dr. Reed Brockway Bontecou, of Troy, N. Y., died at his home in that city March 28, 1907, after an illness of one week, aged 82 years. He graduated in medicine at the Castleton Medical College, Vt., in 1847. He was one of the noted field surgeons during the civil war; was in charge of the Haverwood United States General Hospital, Washington, from 1863 until its discontinuance in 1866; and was breveted lieutenant-colonel and colonel of volunteers in 1865, for faithful and meritorious service during the war. Dr. Bontecou was a frequent contributor to medical literature, especially in the line of military surgery; was a member of the American Medical Association and delegate to the International Medical Congresses of 1887 and 1890. At the time of his death he held, with other positions, that of surgeon to the Marshall Sanitarium, Troy.

